# Isolation of a Novel Beta-2 Human Papillomavirus from Skin

**DOI:** 10.1128/MRA.01628-18

**Published:** 2019-02-28

**Authors:** Rosario N. Brancaccio, Alexis Robitaille, Sankhadeep Dutta, Dana E. Rollison, Massimo Tommasino, Tarik Gheit

**Affiliations:** a Infections and Cancer Biology Group, International Agency for Research on Cancer, Lyon, France; b Department of Cancer Epidemiology, Moffitt Cancer Center, Tampa, Florida, USA; KU Leuven

## Abstract

We report the complete genome characterization of a novel human papillomavirus (HPV) (ICB2) isolated from a skin swab. The L1 region of HPV ICB2 shares 87.9% nucleotide similarity with its closest relative, HPV37, and thus constitutes a novel human betapapillomavirus.

## ANNOUNCEMENT

Human papillomaviruses (HPVs) are double-stranded circular DNA viruses with a genome of approximately 8 kb and belong to the *Papillomaviridae* family. HPVs infect basal keratinocytes of the mucosal and cutaneous epithelia. Based on the nucleotide sequences of the major capsid protein L1, HPVs are classified into five major genera, Alphapapillomavirus, Betapapillomavirus, Gammapapillomavirus, Mupapillomavirus, and Nupapillomavirus (1–3). The mucosal high-risk HPV types, which belong to the genus Alphapapillomavirus, are the etiological agents of anogenital cancers and of a subset of head and neck cancers (4). Moreover, an etiological role of cutaneotrophic HPVs from the genus Betapapillomavirus in association with exposure to UV radiation in the development of nonmelanoma skin cancer is also suggested (5–7).

Here, we report the complete genome sequence of a novel HPV type (HPV ICB2; 7,441 bp) isolated from a human forearm skin swab.

A partial L1 region sequence of HPV ICB2 (99 bp) was previously obtained from DNA extracted from the skin swab using broad-spectrum primers in combination with next-generation sequencing (8). Multiply primed rolling-circle amplification (RCA) was performed on the corresponding skin swab DNA according to the manufacturer’s instructions (illustra TempliPhi 100 amplification kit; GE Healthcare, USA). To obtain the complete viral genome, first, long-range PCR was performed on the RCA product using PrimeSTAR GXL DNA polymerase (TaKaRa Bio), outward-directed primers specific for HPV ICB2 (forward primer, 5′-CAGACAGAACACATCTTTTGATCC-3′; and reverse primer, 5′-TCGTCCCGTGACCCACCCTGA-3′).

The resulting amplicon of approximately 8 kb was then cloned in pCR-XL-2 TOPO vector using the TOPO XL-2 complete PCR cloning kit (Invitrogen, Carlsbad, CA). The sequence of the whole genome was obtained by Sanger sequencing using a primer-walking strategy (GATC Biotech, Germany). This sequencing service uses cycle sequencing technology (dideoxy chain termination/cycle sequencing) on an ABI 3730XL sequencing machine. The viral genome was covered at least twice in order to identify and correct sequencing errors. Thirty-one sequences were generated and aligned to reconstruct the whole genome using the CAP3 sequence assembly program (9), with default parameters.

The clone has been submitted to the International Human Papillomavirus Reference Center in Stockholm (www.hpvcenter.se) for assignment of HPV type number.

The L1 open reading frame (ORF) of HPV ICB2 showed 87.9% nucleotide identity with its closest relative, HPV37, which belongs to the species beta-2 of the genus Betapapillomavirus. HPV ICB2 thus constitutes a novel human betapapillomavirus by sharing less than 90% nucleotide sequence identity with the closest HPV type in the L1 ORF (3).

The G+C content of ICB2 is 40.7%. The virus has the typical genome organization of other cutaneotrophic HPVs; it is composed of five early (E1, E2, E4, E6, and E7) and two late (L1 and L2) ORFs, and no E5 was identified.

The long control region (LCR) is 382 bp. This region contains two polyadenylation sites (AATAAA) for L1 and L2 transcripts and four consensus palindromic E2-binding sites, as follows: ACCG-N_4_-CGGT (*n = 2*), ACC-N_5_-GGT (*n = 1*), and ACC-N_1_-GGT (*n = 1*). A putative TATA box domain (TATAAGA) for the downstream early promoter was also identified.

The two conserved zinc-binding domains of the viral E6 protein [CxxC(x)_29_CxxC and CxxC(x)_30_CxxC] are present and are separated by 36 amino acids (5).

A zinc-binding domain [CxxC(x)_29_CxxC] and one LxCxE motif are located in the E7 protein (5). An ATP-binding site (GPPDTGKS) for ATP-dependent helicase activity was identified in the carboxy terminus of the E1 protein. In conclusion, we identified and fully characterized a new HPV belonging to species beta-2, HPV ICB2. This finding contributes to the expansion of our knowledge about the impressive diversity of the Betapapillomavirus genus.

### Data availability.

The complete genome sequence of HPV ICB2 is available in GenBank under accession number MK080568.
